# μ-Acetato-diacetato{μ-6,6′-dimethoxy-2,2′-[*o*-phenylenebis(nitrilomethanylylidene)]diphenolato}gadolinium(III)zinc

**DOI:** 10.1107/S160053681102890X

**Published:** 2011-08-02

**Authors:** Fan Yang, Guang-Ming Li, Peng Chen, Peng-Fei Yan, Guang-Feng Hou

**Affiliations:** a Key Laboratory of Functional Inorganic Materials Chemistry (MOE), School of Chemistry and Materials Science, Heilongjiang University, Harbin, 150080, People’s Republic of China

## Abstract

In the heterodinuclear title complex, [GdZn(C_22_H_18_N_2_O_4_)(CH_3_COO)_3_], the Zn^II^ ion is five-coordinated in a square-pyramidal environment defined by two O atoms and two N atoms from the ligand, forming the square plane, and one acetate O atom serving as the apex, while the Gd^III^ ion is nine-coordinated in an approximate mono-capped tetra­gonal–anti­prismatic environment defined by four O atoms from the ligand and five acetate O atoms.

## Related literature

For the synthesis of the ligand, see: Costes *et al.* (2000 ▶). For similar 3*d*–4*f* complexes of similar ligands, see: Bao *et al.* (2010 ▶); Liao *et al.* (2010 ▶); Xu *et al.* (2011 ▶).
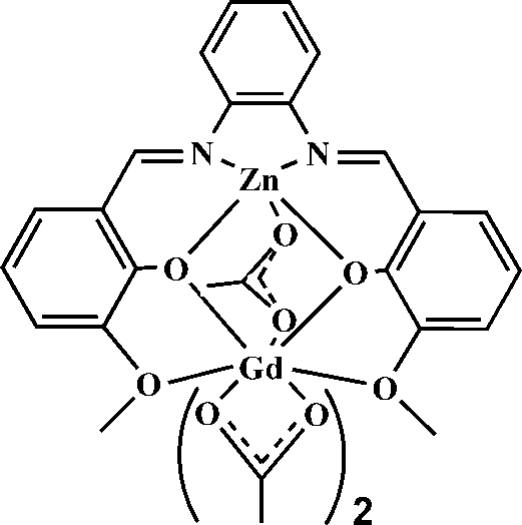


## Experimental

### 

#### Crystal data

[GdZn(C_22_H_18_N_2_O_4_)(C_2_H_3_O_2_)_3_]
*M* *_r_* = 774.16
Monoclinic, 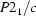

*a* = 14.012 (3) Å
*b* = 13.581 (3) Å
*c* = 15.426 (3) Å
β = 103.65 (3)°
*V* = 2852.6 (10) Å^3^
*Z* = 4
Mo *K*α radiation
μ = 3.21 mm^−1^
*T* = 293 K
0.15 × 0.14 × 0.13 mm

#### Data collection

Rigaku R-AXIS RAPID diffractometer
Absorption correction: multi-scan (*ABSCOR*; Higashi, 1995 ▶) *T*_min_ = 0.645, *T*_max_ = 0.681
26483 measured reflections
6488 independent reflections
4513 reflections with *I* > 2σ(*I*)
*R*_int_ = 0.073

#### Refinement

*R*[*F* ^2^ > 2σ(*F* ^2^)] = 0.041
*wR*(*F* ^2^) = 0.078
*S* = 1.01
6488 reflections
384 parameters
H-atom parameters constrained
Δρ_max_ = 0.59 e Å^−3^
Δρ_min_ = −0.82 e Å^−3^

### 

Data collection: *RAPID-AUTO* (Rigaku, 1998 ▶); cell refinement: *RAPID-AUTO*; data reduction: *CrystalClear* (Rigaku/MSC, 2002 ▶); program(s) used to solve structure: *SHELXS97* (Sheldrick, 2008 ▶); program(s) used to refine structure: *SHELXL97* (Sheldrick, 2008 ▶); molecular graphics: *SHELXTL* (Sheldrick, 2008 ▶); software used to prepare material for publication: *SHELXL97*.

## Supplementary Material

Crystal structure: contains datablock(s) I, global. DOI: 10.1107/S160053681102890X/ng5198sup1.cif

Structure factors: contains datablock(s) I. DOI: 10.1107/S160053681102890X/ng5198Isup2.hkl

Additional supplementary materials:  crystallographic information; 3D view; checkCIF report

## Figures and Tables

**Fig. 1.**
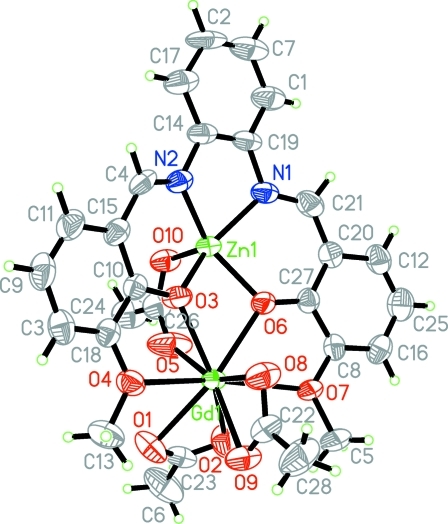
The molecular structure of the title compound, showing 50% probability displacement ellipsoids.

## supplementary crystallographic information

### Comment

Lanthanide complexes with spectroscopic and magnetic properties are currently of considerable interest. In continuation of our studies of salen-type lanthanide complexes (Bao *et al.*, 2010, Xu *et al.*, 2011), we present here the synthesis and the crystal structure of the title compound.

In the title compound,the Zn(II) ion is five-coordinated by two imino nitrogen atoms and two phenolate oxygen atoms from the ligand, and one oxygen from the bridging acetate group to form the pyramid coordination geometry. The Zn—N bond distances are in the range of 2.042 (4) Å—2.062 (4) Å, and the Zn—O bond distances are in the range of 1.974 (3) Å—2.004 (3) Å, which in accordance with the reported values (Liao *et al.* 2010). The Gd(III) ion is ligated to five oxygen atoms of three acetate groups, and four oxygen atoms from the ligand. The Gd—O bond distances are in the range of 2.365 (3)—2.626 (3) Å (Fig.1, Table 1). The Gd—Zn distance is 3.414 (1) Å. The positive charge of the Gd(III) and Zn(II) ions are balanced by the ligand *L*^2-^ and three acetate groups (*L* = *N*,*N*'-bis(2-oxy-3-methoxybenzylidene)- 1,2-diaminobenzene).

### Experimental

The salen lignad was synthesized following the reference (Costes *et al.* 2000). To a 1:1 MeOH/CH_2_Cl_2_ solution (20 ml) of H_2_L (0.0748 g, 0.2 mmol) andGd(CH_3_COO)_3_˙4H_2_O (0.0813 g, 0.2 mmol) was added a MeOH solution (5 ml) ofZn(CH_3_COO)_2_˙2H_2_O (0.0438 g, 0.2 mmol) at the ambient temperature. The color of the solution immediately changed to yellow. After stirring for 8 h, the solution was filtered to remove the suspended particles. yellow single crystals suitable for X-ray diffraction were obtained by slow diffusion of diethylether into the filtrate in four days. ZnGd(C_22_H_18_N_2_O_4_)(CH_3_COO)_3_, Elemental Anal. Calc.: C, 43.44; H, 3.52; N, 3.62 wt%, Found: C, 43.38; H, 3.44; N, 3.27 wt%.

### Refinement

The anormal reflection data (-1 1 4) have been omitted during the refinement. H atoms bound to C atoms were placed in calculated positions and treated as riding on their parent atoms, with C—H = 0.93 Å (aromatic C) and *U*_iso_(H) = 1.2Ueq(C), and with C—H = 0.96 Å (methyl C) and *U*_iso_(H) = 1.5Ueq(C).

### Crystal data

**Table:**

[GdZn(C_22_H_18_N_2_O_4_)(C_2_H_3_O_2_)_3_]	*F*(000) = 1532
*M**_r_* = 774.16	*D*_x_ = 1.803 Mg m^−^^3^
Monoclinic, *P*2_1_/*c*	Mo *K*α radiation, λ = 0.71073 Å
Hall symbol: -P 2ybc	Cell parameters from 16409 reflections
*a* = 14.012 (3) Å	θ = 3.0–27.5°
*b* = 13.581 (3) Å	µ = 3.21 mm^−^^1^
*c* = 15.426 (3) Å	*T* = 293 K
β = 103.65 (3)°	Block, yellow
*V* = 2852.6 (10) Å^3^	0.15 × 0.14 × 0.13 mm
*Z* = 4	

### Data collection

**Table:**

Rigaku R-AXIS RAPID diffractometer	6488 independent reflections
Radiation source: fine-focus sealed tube	4513 reflections with *I* > 2σ(*I*)
graphite	*R*_int_ = 0.073
ω scan	θ_max_ = 27.5°, θ_min_ = 3.0°
Absorption correction: multi-scan (*ABSCOR*; Higashi, 1995)	*h* = −18→18
*T*_min_ = 0.645, *T*_max_ = 0.681	*k* = −17→16
26483 measured reflections	*l* = −19→18

### Refinement

**Table:**

Refinement on *F*^2^	Primary atom site location: structure-invariant direct methods
Least-squares matrix: full	Secondary atom site location: difference Fourier map
*R*[*F*^2^ > 2σ(*F*^2^)] = 0.041	Hydrogen site location: inferred from neighbouring sites
*wR*(*F*^2^) = 0.078	H-atom parameters constrained
*S* = 1.01	*w* = 1/[σ^2^(*F*_o_^2^) + (0.0272*P*)^2^ + 1.1216*P*] where *P* = (*F*_o_^2^ + 2*F*_c_^2^)/3
6488 reflections	(Δ/σ)_max_ < 0.001
384 parameters	Δρ_max_ = 0.59 e Å^−^^3^
0 restraints	Δρ_min_ = −0.82 e Å^−^^3^

### Special details

**Table:**

Geometry. All e.s.d.'s (except the e.s.d. in the dihedral angle between two l.s. planes) are estimated using the full covariance matrix. The cell e.s.d.'s are taken into account individually in the estimation of e.s.d.'s in distances, angles and torsion angles; correlations between e.s.d.'s in cell parameters are only used when they are defined by crystal symmetry. An approximate (isotropic) treatment of cell e.s.d.'s is used for estimating e.s.d.'s involving l.s. planes.
Refinement. Refinement of *F*^2^ against ALL reflections. The weighted *R*-factor *wR* and goodness of fit *S* are based on *F*^2^, conventional *R*-factors *R* are based on *F*, with *F* set to zero for negative *F*^2^. The threshold expression of *F*^2^ > σ(*F*^2^) is used only for calculating *R*-factors(gt) *etc*. and is not relevant to the choice of reflections for refinement. *R*-factors based on *F*^2^ are statistically about twice as large as those based on *F*, and *R*- factors based on ALL data will be even larger.

### Fractional atomic coordinates and isotropic or equivalent isotropic displacement parameters (Å^2^)

**Table:**

	*x*	*y*	*z*	*U*_iso_*/*U*_eq_	
Gd1	0.298502 (17)	0.880640 (16)	0.188340 (16)	0.03607 (7)	
Zn1	0.05908 (4)	0.84037 (4)	0.19232 (4)	0.03815 (14)	
O1	0.4228 (3)	0.9767 (3)	0.2964 (3)	0.0660 (12)	
O2	0.4373 (3)	0.8188 (3)	0.3022 (3)	0.0696 (12)	
O3	0.1371 (2)	0.9446 (2)	0.1482 (2)	0.0457 (8)	
O4	0.2720 (3)	1.0662 (2)	0.1405 (2)	0.0469 (8)	
O5	0.2482 (3)	0.8884 (4)	0.3247 (3)	0.0844 (14)	
O6	0.1715 (2)	0.7605 (2)	0.1712 (2)	0.0448 (9)	
O7	0.3403 (2)	0.6960 (2)	0.1719 (2)	0.0459 (9)	
O8	0.2722 (3)	0.8700 (3)	0.0262 (2)	0.0581 (10)	
O9	0.4224 (3)	0.8956 (3)	0.1017 (3)	0.0594 (10)	
O10	0.0909 (3)	0.8682 (2)	0.3216 (2)	0.0495 (9)	
N1	−0.0301 (3)	0.7181 (3)	0.1660 (3)	0.0426 (10)	
N2	−0.0693 (3)	0.9076 (3)	0.1312 (3)	0.0406 (9)	
C1	−0.2061 (4)	0.6805 (5)	0.1540 (4)	0.0594 (15)	
H1A	−0.1919	0.6148	0.1686	0.071*	
C2	−0.3228 (4)	0.8097 (6)	0.1166 (4)	0.0722 (18)	
H2A	−0.3877	0.8308	0.1047	0.087*	
C3	0.1527 (5)	1.1987 (3)	0.0881 (3)	0.0528 (14)	
H3A	0.2012	1.2443	0.0847	0.063*	
C4	−0.0760 (4)	0.9959 (3)	0.0987 (3)	0.0441 (12)	
H4A	−0.1388	1.0190	0.0729	0.053*	
C5	0.4366 (4)	0.6643 (4)	0.1674 (5)	0.0705 (18)	
H5A	0.4329	0.6302	0.1122	0.106*	
H5B	0.4626	0.6208	0.2164	0.106*	
H5C	0.4788	0.7205	0.1707	0.106*	
C6	0.5288 (6)	0.9052 (5)	0.4231 (4)	0.095 (3)	
H6A	0.5924	0.8835	0.4180	0.142*	
H6B	0.5060	0.8634	0.4642	0.142*	
H6C	0.5331	0.9718	0.4445	0.142*	
C7	−0.3015 (5)	0.7124 (5)	0.1369 (4)	0.0728 (18)	
H7A	−0.3517	0.6683	0.1390	0.087*	
C8	0.2713 (4)	0.6248 (3)	0.1765 (3)	0.0399 (11)	
C9	0.0535 (5)	1.2258 (4)	0.0651 (4)	0.0602 (16)	
H9A	0.0361	1.2898	0.0466	0.072*	
C10	0.1059 (4)	1.0339 (3)	0.1223 (3)	0.0373 (11)	
C11	−0.0183 (4)	1.1587 (4)	0.0697 (4)	0.0550 (15)	
H11A	−0.0838	1.1777	0.0532	0.066*	
C12	0.1196 (5)	0.4954 (3)	0.1731 (4)	0.0541 (14)	
H12A	0.0680	0.4512	0.1691	0.065*	
C13	0.3513 (4)	1.1311 (4)	0.1360 (4)	0.0594 (15)	
H13A	0.3416	1.1567	0.0765	0.089*	
H13B	0.4122	1.0956	0.1513	0.089*	
H13C	0.3531	1.1845	0.1771	0.089*	
C14	−0.1514 (4)	0.8451 (4)	0.1298 (3)	0.0459 (12)	
C15	0.0052 (4)	1.0620 (3)	0.0987 (3)	0.0426 (12)	
C16	0.2889 (4)	0.5246 (3)	0.1824 (3)	0.0512 (13)	
H16A	0.3522	0.5005	0.1880	0.061*	
C17	−0.2486 (4)	0.8776 (5)	0.1134 (4)	0.0600 (14)	
H17A	−0.2638	0.9435	0.1005	0.072*	
C18	0.1774 (4)	1.1038 (3)	0.1156 (3)	0.0395 (11)	
C19	−0.1301 (4)	0.7453 (4)	0.1496 (3)	0.0457 (12)	
C20	0.0996 (4)	0.5986 (3)	0.1719 (3)	0.0424 (12)	
C21	−0.0006 (4)	0.6290 (4)	0.1625 (3)	0.0510 (13)	
H21A	−0.0482	0.5798	0.1531	0.061*	
C22	0.3616 (4)	0.8806 (4)	0.0284 (4)	0.0524 (13)	
C23	0.4599 (4)	0.9003 (4)	0.3355 (4)	0.0498 (13)	
C24	0.2012 (5)	0.8960 (5)	0.4602 (4)	0.0749 (18)	
H24A	0.2498	0.9466	0.4778	0.112*	
H24B	0.2257	0.8352	0.4886	0.112*	
H24C	0.1423	0.9144	0.4777	0.112*	
C25	0.2112 (5)	0.4601 (4)	0.1798 (4)	0.0632 (16)	
H25A	0.2223	0.3925	0.1827	0.076*	
C26	0.1791 (4)	0.8833 (4)	0.3610 (3)	0.0516 (13)	
C27	0.1785 (4)	0.6639 (3)	0.1738 (3)	0.0385 (11)	
C28	0.3968 (5)	0.8751 (5)	−0.0560 (4)	0.084 (2)	
H28A	0.4423	0.9277	−0.0570	0.125*	
H28B	0.3419	0.8811	−0.1064	0.125*	
H28C	0.4289	0.8131	−0.0586	0.125*	

### Atomic displacement parameters (Å^2^)

**Table:**

	*U*^11^	*U*^22^	*U*^33^	*U*^12^	*U*^13^	*U*^23^
Gd1	0.02645 (12)	0.03976 (12)	0.04177 (14)	−0.00167 (10)	0.00757 (9)	−0.00027 (11)
Zn1	0.0263 (3)	0.0438 (3)	0.0441 (3)	0.0004 (2)	0.0079 (2)	−0.0002 (2)
O1	0.071 (3)	0.054 (2)	0.065 (3)	−0.028 (2)	0.000 (2)	0.0017 (19)
O2	0.068 (3)	0.062 (2)	0.064 (3)	0.008 (2)	−0.014 (2)	−0.0066 (19)
O3	0.030 (2)	0.0417 (18)	0.065 (2)	0.0033 (14)	0.0124 (17)	0.0161 (15)
O4	0.041 (2)	0.0432 (18)	0.057 (2)	−0.0066 (15)	0.0117 (17)	0.0041 (15)
O5	0.045 (3)	0.166 (4)	0.044 (2)	−0.021 (3)	0.014 (2)	−0.010 (3)
O6	0.034 (2)	0.0337 (18)	0.070 (3)	−0.0015 (14)	0.0186 (18)	−0.0013 (15)
O7	0.0256 (19)	0.0440 (18)	0.066 (2)	0.0072 (14)	0.0074 (16)	−0.0030 (15)
O8	0.041 (2)	0.090 (3)	0.044 (2)	0.004 (2)	0.0114 (17)	−0.0026 (18)
O9	0.036 (2)	0.087 (3)	0.057 (3)	−0.0017 (19)	0.0137 (19)	0.001 (2)
O10	0.036 (2)	0.070 (2)	0.043 (2)	0.0000 (17)	0.0112 (16)	0.0014 (16)
N1	0.031 (2)	0.054 (2)	0.044 (3)	−0.0075 (18)	0.0116 (19)	−0.0008 (18)
N2	0.033 (2)	0.051 (2)	0.037 (2)	0.0069 (17)	0.0061 (18)	−0.0012 (17)
C1	0.041 (4)	0.088 (4)	0.051 (4)	−0.013 (3)	0.016 (3)	0.000 (3)
C2	0.022 (3)	0.134 (6)	0.061 (4)	−0.009 (3)	0.010 (3)	−0.007 (4)
C3	0.070 (4)	0.039 (3)	0.047 (3)	0.000 (3)	0.008 (3)	0.002 (2)
C4	0.034 (3)	0.062 (3)	0.036 (3)	0.017 (2)	0.008 (2)	−0.005 (2)
C5	0.028 (3)	0.069 (4)	0.112 (6)	0.011 (3)	0.014 (3)	−0.010 (3)
C6	0.096 (6)	0.110 (5)	0.064 (5)	−0.045 (5)	−0.008 (4)	−0.005 (4)
C7	0.040 (4)	0.110 (5)	0.068 (5)	−0.022 (3)	0.012 (3)	0.000 (4)
C8	0.046 (3)	0.036 (2)	0.036 (3)	0.001 (2)	0.007 (2)	−0.0035 (19)
C9	0.078 (5)	0.038 (3)	0.060 (4)	0.014 (3)	0.008 (3)	0.001 (2)
C10	0.040 (3)	0.038 (2)	0.032 (3)	0.006 (2)	0.007 (2)	−0.0005 (18)
C11	0.050 (4)	0.050 (3)	0.058 (4)	0.017 (3)	0.000 (3)	−0.002 (2)
C12	0.067 (4)	0.041 (3)	0.057 (4)	−0.011 (3)	0.020 (3)	0.001 (2)
C13	0.051 (4)	0.063 (3)	0.063 (4)	−0.017 (3)	0.010 (3)	0.012 (3)
C14	0.028 (3)	0.069 (3)	0.039 (3)	−0.001 (2)	0.005 (2)	−0.005 (2)
C15	0.042 (3)	0.048 (3)	0.036 (3)	0.011 (2)	0.005 (2)	−0.002 (2)
C16	0.058 (4)	0.046 (3)	0.050 (3)	0.015 (3)	0.014 (3)	0.006 (2)
C17	0.038 (3)	0.090 (4)	0.050 (3)	0.007 (3)	0.007 (3)	−0.002 (3)
C18	0.045 (3)	0.040 (3)	0.031 (3)	0.004 (2)	0.006 (2)	−0.0037 (18)
C19	0.029 (3)	0.066 (3)	0.041 (3)	−0.010 (2)	0.007 (2)	−0.004 (2)
C20	0.050 (3)	0.041 (3)	0.038 (3)	−0.005 (2)	0.016 (2)	0.0008 (19)
C21	0.054 (4)	0.051 (3)	0.051 (3)	−0.017 (3)	0.018 (3)	0.000 (2)
C22	0.046 (3)	0.059 (3)	0.056 (4)	0.009 (3)	0.020 (3)	0.002 (3)
C23	0.036 (3)	0.066 (4)	0.047 (3)	−0.015 (3)	0.008 (2)	0.002 (3)
C24	0.067 (5)	0.114 (5)	0.043 (4)	−0.003 (4)	0.011 (3)	−0.002 (3)
C25	0.079 (5)	0.041 (3)	0.071 (4)	0.004 (3)	0.019 (4)	0.006 (3)
C26	0.049 (4)	0.066 (3)	0.038 (3)	0.001 (3)	0.006 (3)	−0.001 (2)
C27	0.044 (3)	0.035 (2)	0.038 (3)	−0.001 (2)	0.013 (2)	0.0001 (19)
C28	0.078 (5)	0.120 (5)	0.061 (4)	0.023 (4)	0.032 (4)	−0.006 (4)

### Geometric parameters (Å, °)

**Table:**

Gd1—O3	2.365 (3)	C4—C15	1.449 (7)
Gd1—O5	2.373 (4)	C4—H4A	0.9300
Gd1—O6	2.382 (3)	C5—H5A	0.9600
Gd1—O9	2.436 (4)	C5—H5B	0.9600
Gd1—O2	2.441 (4)	C5—H5C	0.9600
Gd1—O8	2.445 (4)	C6—C23	1.465 (8)
Gd1—O1	2.479 (4)	C6—H6A	0.9600
Gd1—O7	2.601 (3)	C6—H6B	0.9600
Gd1—O4	2.627 (3)	C6—H6C	0.9600
Gd1—C22	2.810 (6)	C7—H7A	0.9300
Gd1—C23	2.814 (5)	C8—C16	1.382 (6)
Gd1—Zn1	3.4139 (9)	C8—C27	1.395 (6)
Zn1—O10	1.975 (3)	C9—C11	1.371 (8)
Zn1—O6	2.002 (3)	C9—H9A	0.9300
Zn1—O3	2.003 (3)	C10—C18	1.402 (6)
Zn1—N2	2.039 (4)	C10—C15	1.425 (6)
Zn1—N1	2.060 (4)	C11—C15	1.401 (6)
O1—C23	1.250 (6)	C11—H11A	0.9300
O2—C23	1.230 (6)	C12—C25	1.351 (8)
O3—C10	1.319 (5)	C12—C20	1.429 (6)
O4—C18	1.387 (6)	C12—H12A	0.9300
O4—C13	1.434 (6)	C13—H13A	0.9600
O5—C26	1.230 (7)	C13—H13B	0.9600
O6—C27	1.315 (5)	C13—H13C	0.9600
O7—C8	1.382 (5)	C14—C17	1.397 (7)
O7—C5	1.433 (6)	C14—C19	1.406 (7)
O8—C22	1.253 (6)	C16—C25	1.390 (8)
O9—C22	1.261 (6)	C16—H16A	0.9300
O10—C26	1.259 (6)	C17—H17A	0.9300
N1—C21	1.284 (6)	C20—C27	1.413 (6)
N1—C19	1.413 (6)	C20—C21	1.438 (7)
N2—C4	1.295 (6)	C21—H21A	0.9300
N2—C14	1.427 (6)	C22—C28	1.499 (8)
C1—C7	1.370 (8)	C24—C26	1.498 (7)
C1—C19	1.396 (7)	C24—H24A	0.9600
C1—H1A	0.9300	C24—H24B	0.9600
C2—C7	1.375 (8)	C24—H24C	0.9600
C2—C17	1.398 (8)	C25—H25A	0.9300
C2—H2A	0.9300	C28—H28A	0.9600
C3—C18	1.376 (6)	C28—H28B	0.9600
C3—C9	1.400 (8)	C28—H28C	0.9600
C3—H3A	0.9300		
			
O3—Gd1—O5	75.50 (14)	C7—C1—C19	120.8 (6)
O3—Gd1—O6	65.12 (10)	C7—C1—H1A	119.6
O5—Gd1—O6	76.04 (13)	C19—C1—H1A	119.6
O3—Gd1—O9	125.94 (12)	C7—C2—C17	121.3 (6)
O5—Gd1—O9	151.85 (14)	C7—C2—H2A	119.4
O6—Gd1—O9	127.31 (12)	C17—C2—H2A	119.4
O3—Gd1—O2	149.88 (14)	C18—C3—C9	119.1 (5)
O5—Gd1—O2	74.38 (16)	C18—C3—H3A	120.4
O6—Gd1—O2	106.97 (12)	C9—C3—H3A	120.4
O9—Gd1—O2	82.74 (14)	N2—C4—C15	126.0 (4)
O3—Gd1—O8	81.38 (13)	N2—C4—H4A	117.0
O5—Gd1—O8	154.78 (14)	C15—C4—H4A	117.0
O6—Gd1—O8	85.25 (12)	O7—C5—H5A	109.5
O9—Gd1—O8	53.03 (13)	O7—C5—H5B	109.5
O2—Gd1—O8	128.15 (14)	H5A—C5—H5B	109.5
O3—Gd1—O1	117.37 (13)	O7—C5—H5C	109.5
O5—Gd1—O1	71.90 (14)	H5A—C5—H5C	109.5
O6—Gd1—O1	145.40 (12)	H5B—C5—H5C	109.5
O9—Gd1—O1	81.16 (13)	C23—C6—H6A	109.5
O2—Gd1—O1	51.91 (13)	C23—C6—H6B	109.5
O8—Gd1—O1	129.16 (13)	H6A—C6—H6B	109.5
O3—Gd1—O7	123.52 (10)	C23—C6—H6C	109.5
O5—Gd1—O7	104.50 (14)	H6A—C6—H6C	109.5
O6—Gd1—O7	60.56 (10)	H6B—C6—H6C	109.5
O9—Gd1—O7	79.67 (12)	C1—C7—C2	119.8 (6)
O2—Gd1—O7	65.11 (11)	C1—C7—H7A	120.1
O8—Gd1—O7	79.98 (11)	C2—C7—H7A	120.1
O1—Gd1—O7	115.73 (12)	O7—C8—C16	125.2 (5)
O3—Gd1—O4	60.72 (10)	O7—C8—C27	113.0 (4)
O5—Gd1—O4	98.87 (14)	C16—C8—C27	121.8 (5)
O6—Gd1—O4	124.96 (11)	C11—C9—C3	120.6 (5)
O9—Gd1—O4	80.67 (12)	C11—C9—H9A	119.7
O2—Gd1—O4	124.60 (12)	C3—C9—H9A	119.7
O8—Gd1—O4	78.02 (11)	O3—C10—C18	117.0 (4)
O1—Gd1—O4	73.41 (12)	O3—C10—C15	124.1 (4)
O7—Gd1—O4	156.55 (11)	C18—C10—C15	118.8 (4)
O3—Gd1—C22	104.47 (14)	C9—C11—C15	121.3 (5)
O5—Gd1—C22	177.28 (16)	C9—C11—H11A	119.3
O6—Gd1—C22	106.46 (15)	C15—C11—H11A	119.3
O9—Gd1—C22	26.62 (14)	C25—C12—C20	121.9 (5)
O2—Gd1—C22	105.62 (16)	C25—C12—H12A	119.0
O8—Gd1—C22	26.43 (14)	C20—C12—H12A	119.0
O1—Gd1—C22	105.89 (16)	O4—C13—H13A	109.5
O7—Gd1—C22	77.82 (13)	O4—C13—H13B	109.5
O4—Gd1—C22	78.86 (13)	H13A—C13—H13B	109.5
O3—Gd1—C23	135.50 (14)	O4—C13—H13C	109.5
O5—Gd1—C23	68.36 (15)	H13A—C13—H13C	109.5
O6—Gd1—C23	126.30 (13)	H13B—C13—H13C	109.5
O9—Gd1—C23	83.90 (14)	C17—C14—C19	119.5 (5)
O2—Gd1—C23	25.85 (13)	C17—C14—N2	124.3 (5)
O8—Gd1—C23	136.84 (14)	C19—C14—N2	116.2 (4)
O1—Gd1—C23	26.35 (12)	C11—C15—C10	118.4 (5)
O7—Gd1—C23	90.78 (13)	C11—C15—C4	117.1 (5)
O4—Gd1—C23	99.73 (13)	C10—C15—C4	124.4 (4)
C22—Gd1—C23	110.42 (16)	C8—C16—C25	119.5 (5)
O3—Gd1—Zn1	34.95 (7)	C8—C16—H16A	120.3
O5—Gd1—Zn1	59.49 (10)	C25—C16—H16A	120.3
O6—Gd1—Zn1	35.01 (7)	C14—C17—C2	119.1 (6)
O9—Gd1—Zn1	148.60 (9)	C14—C17—H17A	120.5
O2—Gd1—Zn1	123.47 (11)	C2—C17—H17A	120.5
O8—Gd1—Zn1	95.59 (10)	C3—C18—O4	125.9 (5)
O1—Gd1—Zn1	127.58 (10)	C3—C18—C10	121.7 (5)
O7—Gd1—Zn1	95.30 (7)	O4—C18—C10	112.5 (4)
O4—Gd1—Zn1	94.82 (8)	C1—C19—C14	119.5 (5)
C22—Gd1—Zn1	121.98 (13)	C1—C19—N1	124.2 (5)
C23—Gd1—Zn1	127.37 (11)	C14—C19—N1	116.2 (4)
O10—Zn1—O6	105.65 (14)	C27—C20—C12	117.8 (5)
O10—Zn1—O3	101.36 (14)	C27—C20—C21	124.3 (4)
O6—Zn1—O3	79.28 (13)	C12—C20—C21	117.8 (5)
O10—Zn1—N2	110.22 (15)	N1—C21—C20	125.6 (5)
O6—Zn1—N2	144.04 (15)	N1—C21—H21A	117.2
O3—Zn1—N2	91.09 (15)	C20—C21—H21A	117.2
O10—Zn1—N1	109.55 (15)	O8—C22—O9	120.1 (5)
O6—Zn1—N1	89.71 (14)	O8—C22—C28	120.2 (5)
O3—Zn1—N1	148.98 (15)	O9—C22—C28	119.7 (5)
N2—Zn1—N1	80.92 (16)	O8—C22—Gd1	60.3 (3)
O10—Zn1—Gd1	89.96 (10)	O9—C22—Gd1	59.9 (3)
O6—Zn1—Gd1	43.06 (9)	C28—C22—Gd1	177.1 (4)
O3—Zn1—Gd1	42.55 (9)	O2—C23—O1	120.5 (5)
N2—Zn1—Gd1	133.10 (12)	O2—C23—C6	118.3 (5)
N1—Zn1—Gd1	132.72 (12)	O1—C23—C6	121.1 (5)
C23—O1—Gd1	92.0 (3)	O2—C23—Gd1	59.9 (3)
C23—O2—Gd1	94.3 (3)	O1—C23—Gd1	61.7 (3)
C10—O3—Zn1	125.8 (3)	C6—C23—Gd1	167.8 (5)
C10—O3—Gd1	130.2 (3)	C26—C24—H24A	109.5
Zn1—O3—Gd1	102.50 (12)	C26—C24—H24B	109.5
C18—O4—C13	117.4 (4)	H24A—C24—H24B	109.5
C18—O4—Gd1	119.6 (3)	C26—C24—H24C	109.5
C13—O4—Gd1	122.9 (3)	H24A—C24—H24C	109.5
C26—O5—Gd1	146.3 (4)	H24B—C24—H24C	109.5
C27—O6—Zn1	126.2 (3)	C12—C25—C16	120.1 (5)
C27—O6—Gd1	129.1 (3)	C12—C25—H25A	119.9
Zn1—O6—Gd1	101.93 (12)	C16—C25—H25A	119.9
C8—O7—C5	118.1 (4)	O5—C26—O10	125.3 (5)
C8—O7—Gd1	119.5 (3)	O5—C26—C24	117.6 (5)
C5—O7—Gd1	122.1 (3)	O10—C26—C24	117.0 (5)
C22—O8—Gd1	93.3 (3)	O6—C27—C8	116.3 (4)
C22—O9—Gd1	93.5 (3)	O6—C27—C20	125.0 (4)
C26—O10—Zn1	118.7 (3)	C8—C27—C20	118.7 (4)
C21—N1—C19	123.6 (4)	C22—C28—H28A	109.5
C21—N1—Zn1	125.6 (4)	C22—C28—H28B	109.5
C19—N1—Zn1	110.7 (3)	H28A—C28—H28B	109.5
C4—N2—C14	124.1 (4)	C22—C28—H28C	109.5
C4—N2—Zn1	124.7 (3)	H28A—C28—H28C	109.5
C14—N2—Zn1	111.2 (3)	H28B—C28—H28C	109.5

## References

[bb1] Bao, Y., Li, G.-M., Yang, F., Yan, P.-F. & Chen, P. (2010). *Acta Cryst.* E**66**, m1379. 10.1107/S1600536810039103 PMC3008985 21588818

[bb2] Costes, J. P., Dahan, F. & Dupuis, A. (2000). *Inorg. Chem.* **39**, 5994–6000. 10.1021/ic000666u 11151500

[bb3] Higashi, T. (1995). *ABSCOR* Rigaku Corporation, Tokyo, Japan.

[bb4] Liao, A., Yang, X. P., Stanley, J. M., Jones, R. A. & Holiday, B. J. (2010). *J. Chem. Crystallogr.* **40**, 1060–1064.

[bb5] Rigaku (1998). *RAPID-AUTO* Rigaku Corporation, Tokyo, Japan.

[bb6] Rigaku/MSC (2002). *CrystalClear* Rigaku/MSC Inc., The Woodlands, Texas, USA.

[bb7] Sheldrick, G. M. (2008). *Acta Cryst.* A**64**, 112–122. 10.1107/S0108767307043930 18156677

[bb8] Xu, L., Li, H.-F., Chen, P. & Yan, P.-F. (2011). *Acta Cryst.* E**67**, m367. 10.1107/S1600536811005253 PMC3051998 21522291

